# Effect of thermocycling on the mechanical properties of permanent composite-based CAD-CAM restorative materials produced by additive and subtractive manufacturing techniques

**DOI:** 10.1186/s12903-024-04016-z

**Published:** 2024-03-14

**Authors:** Tuğba Temizci, Hatice Nalan Bozoğulları

**Affiliations:** Department of Prosthodontics, Faculty of Dentistry, Karamanoğlu Mehmetbey University, Karaman, Turkey

**Keywords:** CAD-CAM, 3D-printing, Milling, Mechanical properties

## Abstract

**Background:**

The aim of the study was to determine and compare the biaxial flexural strength (BFS) and Vickers hardness (VHN) of additive and subtractive manufactured permanent composite-based restorative materials, before and after thermal aging.

**Methods:**

A total of 200 specimens were prepared; 100 disc-shaped specimens (diameter 13 × 1.2 mm) for the BFS test and 100 square specimens (14 × 14 × 2 mm) for the VHN test. The specimens were made from various materials: two subtractive composite-based blocks (Cerasmart 270 [CS], Vita Enamic [VE]), two additive composite-based resins used for two different vat polymerization methods (digital light processing [DLP]; Saremco Print Crowntec [SC] and stereolithography [SLA]; Formlabs Permanent Crown Resin [FP]), and one feldspathic glass-matrix ceramic block (Vita Mark II [VM]) as the control group. Specimens of each material were divided into two subgroups: thermal cycled or non-thermal cycled (*n* = 10). BFS and VHN tests were performed on all groups. Data were analyzed with two-way ANOVA and post hoc Tukey test (α = 0.05).

**Results:**

The type of restorative material used for the specimen had a statistically significant influence on both BFS and VHN values. However, thermal cycling did not affect the BFS and VHN values. After thermal cycling, the results of the BFS test were ranked from best to worst as follows: CS, FP, SC, VE, then VM. For the VHN values, the order from best to worst was as follows: VM, VE, CS, FP, then SC.

**Conclusions:**

3D printed and milled composite groups showed higher BFS than feldspathic ceramics. When the VHN results were examined, it was seen that the 3D resin groups had the lowest VHN values. Furthermore, it was observed that the thermal cycle had no effect on BFS or VHN.

## Background

Computer-aided design and computer-aided manufacturing (CAD-CAM) in the production of prosthetic restorations have become very popular as they save time and labor [1]. Compared to traditional methods, CAD-CAM systems have the advantage of skipping error-prone steps, such as impression, wax modeling, and casting [2]. CAD-CAM systems based on the principles of subtractive manufacturing (milling) produce reliable restorations with accurate dimensions. Conversely, milling systems operate by cutting solid blocks into the desired shape, which can lead to material waste and additional costs for milling tools. Additive manufacturing (3D printing) developed with CAD is more economical than milling techniques in terms of hardware investment and total production costs, which minimizes waste from in the process. 3D printing can also produce more complex structures than milling systems. Thanks to this system, the number of appointments needed for the patient during dental prosthesis production is reduced and the production of replacement prostheses is easier since the digital data is stored [3]. The 3D printing process for dental prostheses is accomplished with layering techniques, such as sintering the powder, depositing molten thermoplastic material, or light-curing the resin [4]. The most commonly employed methods for 3D printing dental prostheses are SLA and DLP [5, 6]. SLA uses a laser to create points, while DLP printers employ a digital projector screen to project the layer's image across the entire platform. With the 3D production method, metal substructures, splints, removable prostheses, and temporary prostheses can all be produced [2, 7].

Indirect aesthetic restorations have gained popularity over time and their use has increased significantly [8]. Ceramics and composites are widely used for veneers, inlays, onlays, and crowns due to their tooth-colored properties. Although ceramics have superior optical properties and a natural tooth-like appearance, they suffer from hardness, brittle fracture, chipping, and wear on opposing dentition [9, 10]. Resin composites are less brittle than ceramics, cause less wear on opposing dentition, and can be easily repaired [11, 12]. However, their color stability is low and wear out faster than ceramic [13]. Recently, resin-matrix CAD-CAM indirect restorative materials have been developed that combine the advantageous properties of both ceramics and composites [14]. Nowadays, the development of 3D printing technologies and materials has resulted in the emergence of new printable permanent composite resins that have been proposed for the production of indirect restorations [15, 16]. However, there is limited knowledge about the long-term behavior of 3D printing of permanent composite materials. Resins used in 3D printers need to maintain a stable liquid consistency; therefore, they are believed to contain fewer inorganic fillers compared with block and disc-shaped materials. The low filler content affects the mechanical properties of the material [16–18].

Dental restorations are exposed to various harmful stimuli, such as temperature changes, chewing, and the effect of fluid (water and saliva) in the oral environment. Thermal cycling, a popular artificial aging method, is used in in vitro studies to simulate the thermal changes that occur in the oral cavity during eating and drinking. Numerous in vitro studies of the mechanical performance of dental composite materials have shown that thermal cycling accelerates the deterioration of the material by significantly reducing the mechanical properties [19, 20].

Recent advancements in digital dentistry have given rise to novel permanent composite-based restorative materials and manufacturing techniques for 3D printing dental restorations. Due to their diversity and increasing application by clinicians, it's also important to understand the characteristics, including mechanical properties, that are essential to these new materials' longevity. There are now studies comparing the mechanical properties of various restorative materials produced by the subtractive manufacturing technique [21, 22]. However, since 3D printing composite-based restorative materials are novel, more laboratory and clinical studies are required. Additionally, there isn’t much research comparing milled permanent composite based materials to 3D printed permanent composite materials that are thermocycling to examine mechanical properties. However, the difference between our study and other mechanical properties studies of indirect composite materials is that while we evaluated 3D printed and milled composite materials with each other, we also compared them with feldspathic ceramic, which is frequently used in the fabrication of indirect restorations.

The aim of this study was to compare the mechanical properties of additive manufactured and subtractive manufactured permanent composite-based CAD-CAM restorative materials, and to evaluate the effect of thermal aging on their mechanical properties. The first hypothesis was that thermal cycling would not affect the BFS or VHN of CAD-CAM restorative materials. The second hypothesis was that the type of material would not affect the BFS or VHN of CAD-CAM restorative materials.

## Materıals and methods

**Fig. 1:**
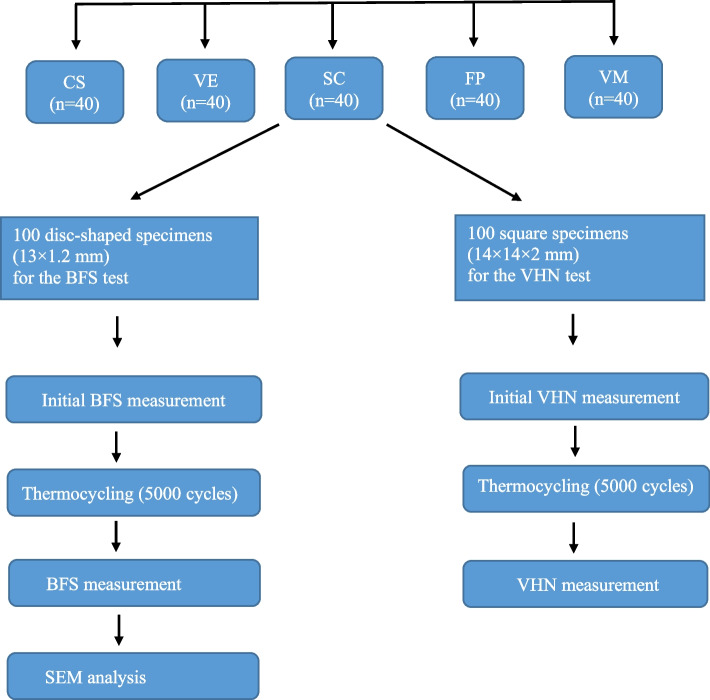
Summary of the study design

In the present study, two different subtractive composite-based CAD-CAM blocks including a polymer-infiltrated ceramic (VE), a hybrid nanoceramic (CS), two different additive composite-based resins used for two different vat polymerization methods (DLP [SC], and SLA [FP]), and one feldspathic glass ceramic (VM; control group) were tested. The properties of materials and manufacturers are presented in Table 1. The study design is presented Fig. 1. A total of 200 specimens were prepared, 40 specimens of each material. For the BFS test, 100 disc-shaped specimens (diameter 13 × 1.2 mm) and for the VHN test, 100 square specimens (14 × 14 × 2 mm) were used (*n* = 20 per material type). Power analysis using G*Power statistical software (G*Power Ver. 3.0.10, Franz Faul, Universität Kiel, Germany) was performed to determine the sample size. A total of 10 samples per group were set considering Power: 0.80, α:0.05, effect size: 2.4 and SD:20, for the tests.

**Table 1:** Materials used in the present study

Material	Abbr	Type	Manufacturer	Manufacturing Technique	Composition
Vita Mark II (control)	VM	Feldspatic glass ceramic	Vita Zahnfabrik,Bad Sackingen, Germany	Milling	20 wt% feldspathic particles with an average particle size of the 4 μm glassy matrix (80 wt %)
Vita Enamic	VE	Polymer infiltrated ceramic	Vita Zahnfabrik,Bad Sackingen, Germany	Milling	14 wt% polymer, 86 wt% feldspar ceramic
Cerasmart 270	CS	Hybrid nanoceramic	GC Corp., Tokyo, Japan	Milling	Nanoparticle-filled resin containing 71 wt% silica and barium glass filler
Saremco print Crowntec	SC	Composite-based resin	Saremco, Dental AG, Switzerland	3D printingDLP	BisEMA % 50 – < 70 Trimethylbenzonyl diphenylphosphine oxide %0.1 – < 1
Formlabs Permanent resin	FP	Composite-based resin	Formlabs Inc., Somerville, MA, USA	3D printingSLA	(Bis-EMA, methacrylate polymer)4′-isopropylidiphenol, ethoxylated and 2- methylprop-2enoic acid Methyl benzoylformate, silanized dental glass, diphenyl (30–50 wt. %—inorganic fillers 2,4,6-trimethylbenzoyl) phosphine oxide, (particle size 0.7 μm)

CAD-CAM milled specimens were sliced from CAD-CAM blocks with a low speed sectioning device (Isomet 1000 Precision Saw, Buehler Lake Bluff, IL USA) using a diamond saw under water cooling for the VHN test. Disc-shaped milled specimens were produced with a 5–axis milling machine (HinriMill 5, Goslar, Germany) from CAD-CAM blocks for the BFS test. 3D models were designed in the Fusion 360 CAD software program (Autodesk, Mill Valley, CA, USA) for both disc-shaped and square 3D printed specimens. These digital designs were exported to Standard Tessellation Language (STL) files for the production of the specimens. SC specimens were printed using a DLP-based 3D printer (MAX UV; ASIGA) with a layer thickness of 50 µm. Following the printing process, the specimens were cleaned with an alcohol-soaked (96%) cloth and then exposed to a post-polymerization process of 4000 lighting exposures with a polymerization device (Otoflash G171; NK Optik, Baierbrunn, Germany). FP samples were printed using the SLA-based 3D printer (Form 3; Formlabs Inc) with a layer thickness of 50 µm. Both 3D printed samples produced with different technologies were printed at 90° orientation for standardization. The printed specimens were washed with 99% isopropyl alcohol for 3 min using ultrasonic cleaning (Form Wash, Formlabs, Somerville, MA, USA) and then exposed to a post-polymerization process using FormCure (Formlabs Inc., Somerville, MA, USA) for 30 min at 60 °C.

The surfaces of all specimens were then ground with a silicon carbide abrasive paper (400-grit and ending with 1200-grit) for 10 secs for each paper under running water. The final thickness of specimens was confirmed using a digital micrometer (Mitutoyo IP65, Mitutoyo Corp., Japan). All specimens were then ultrasonically cleaned in distilled water for 15 min. Specimens for each material group were divided into two subgroups (thermal cycled or nonthermal cycled; *n* = 10 per group) for both BFS and VHN tests.

### Thermal cycling procedure

The specimens of the thermal cycle group of all materials underwent a thermocycling procedure consisting of 5000 cycles in a water bath (of distilled water) ranging from 5 °C to 55 °C. Each cycle lasted 60 secs and consisted of the following steps: 20 secs in a 5 °C bath, 10 secs for transferring the samples to another bath, 20 s in a 55 °C bath, and 10 secs for transferring the samples back to the 5 °C bath. A total of 5,000 cycles at 5 °C and 55 °C were performed, which corresponds to approximately 6 months of clinical use [23].

### Vickers hardness test

Surface hardness was measured with an Emcotest-Durascan G5 hardness testing device (Kuchl, Austria). Five indentations were pressed on the surface of each specimen with a Vickers diamond indenter under a load of 1 kg and a dwell time of 15 secs. The indentation values were obtained using digital processing software. The hardness was computed using the following equation [24]:

$${\text{VHN}}=0.1891\times \left({\text{F}}/{{\text{d}}}^{2}\right),$$

VHN is the Vickers hardness number, F is the applied load expressed in N, and d is the mean length of the two diagonals of the indentation (mm).

### Biaxial flexural strength test

Disc-shaped specimens were assessed with the piston on three-ball test according to ISO 6872 [25], performed in a universal testing machine (Devotrans). The samples were placed on three steel balls with a diameter of 3.4 mm located at the base of the device and placed at an angle of 120° relative to each other. On the upper side of the device, a pressure tip with a diameter of 1.4 mm was placed, which was arranged to contact the sample from the center. A force of 1 mm per min was applied until the pressure tip broke the sample. The force values at the moment when a sample was broken were noted in Newton (N). Since the recorded values are N units, the biaxial bending test result was converted to megapascal (MPa) units using the formula below [26].

$${\text{S}}=-0.2387{\text{P}}\left({\text{X}}-{\text{Y}}\right)/{d}^{2}$$

S=biaxial flexural strength (MPa); P=fracture load (N); d=disc specimen thickness (mm)

$${\text{X}}=\left(1+\upupsilon \right){\text{ln}}\left({\text{B}}/{\text{C}}\right)2+\left[\left(1-\upupsilon \right)/2\right]\left({\text{B}}/{\text{C}}\right)2$$

$${\text{Y}}=\left(1+\upupsilon \right)\left[1+{\text{ln}}\left({\text{A}}/{\text{C}}\right)2\right]+\left(1-\upupsilon \right)\left({\text{A}}/{\text{C}}\right)2$$

υ=Poisson’s ratio; A=radius of the support circle (mm); B=radius of the loaded area (mm); C=radius of the disc specimen (mm)

### Scanning electron microscopy (SEM)

After BFS testing, all fractured specimens were coated with Au and analyzed using scanning electron microscopy (SEM; EVO LS 10; Zeiss, Germany) at 20 kV and 7.4 mm working distance. SEM images were examined at 500 × magnification.

Data were analyzed with two-way ANOVA (IBM SPSS 20.0 software; SPSS Inc., Chicago, IL) and to detect differences among all the groups, Tukey honest post hoc test was used. Statistical significance level was *p* < 0.05. Parametric tests were preferred because the data followed a normal distribution.

## Results

According to the results of the two-way ANOVA, the type of material was found to be significant for BFS and VHN values, while the effect of aging alone was found insignificant. In addition, it was seen that the interaction of material and aging is important for VHN (Table 2).

**Table 2:** Results of two-way ANOVA for biaxial flexural strength and Vickers hardness

Test method	Source of variation	Sum of squares	df	Mean square	F	*p*
Biaxial flexural strength	Material	198073,96	4	49518,49	46,60	0,001
	Aging	687,54	1	687,54	0,64	0,466
	Material x Aging	4250,27	4	1062,56	1,15	0,338
	Error	83121,52	90	923,572		
	Total	4666115,39	100			
Vickers hardness	Material	3542703,27	4	885675,81	536,77	0,000
	Aging	3491,31	1	3491,31	2,11	0,219
	Material x Aging	6599,95	4	1649,98	22,06	0,000
	Error	6728,84	90	74,765		
	Total	3079535,95	100			

*P* < 0,05

Before the thermal cycle, the highest BFS value was with CS (296.11 ± 39.13 MPa), and the lowest value was VM (173.49 ± 22.74 MPa). There was no significant difference between FP and SC groups and VE and VM groups. After the thermal cycle, a general decrease in BFS values was observed in all materials; however, this change was statistically insignificant (Table 3).

**Table 3:** Biaxial flexural strength result (mean ± SD) (MPa)

Material	Non-aging	Aging
CS	296.11 ± 11.56^a^	278.05 ± 6.11^a^
VE	173.99 ± 1.99^c^	173.63 ± 4.77^c^
VM	173.49 ± 3.47^c^	153.49 ± 5.37^c^
SC	232.67 ± 5.94^b^	215.31 ± 6.39^b^
FP	234.67 ± 6.14^b^	230.23 ± 10.35^b^

^*^Different superscript letters in each column indicates statistically significant differences (*p* < 0.05)

When we observed the VHN values before the thermal cycle, the hardest material was VM (548.58 ± 21.94 Hv1). FP and SC were found to have statistically the same hardness values. While the hardness change in VE and VM groups was significant after the thermal cycle, the changes in CS, SC, and FP groups were insignificant (Table 4).

**Table 4:** Vickers hardness test results (mean ± SD) (Hv1)

Material	Non-aging	Aging
CS	89.85 ± 1.12^e^	84.94 ± 0.78^e^
VE	211.80 ± 9.61^c^	203.39 ± 3.10^d^
VM	548.58 ± 11.27^a^	506.05 ± 1.78^b^
SC	25.08 ± 0.45^f^	24.57 ± 0.13^f^
FP	29.39 ± 0.53^f^	28.96 ± 0.47^f^

^*^Different superscript letters in each column indicates statistically significant differences (*p* < 0.05)

**Fig. 2:**
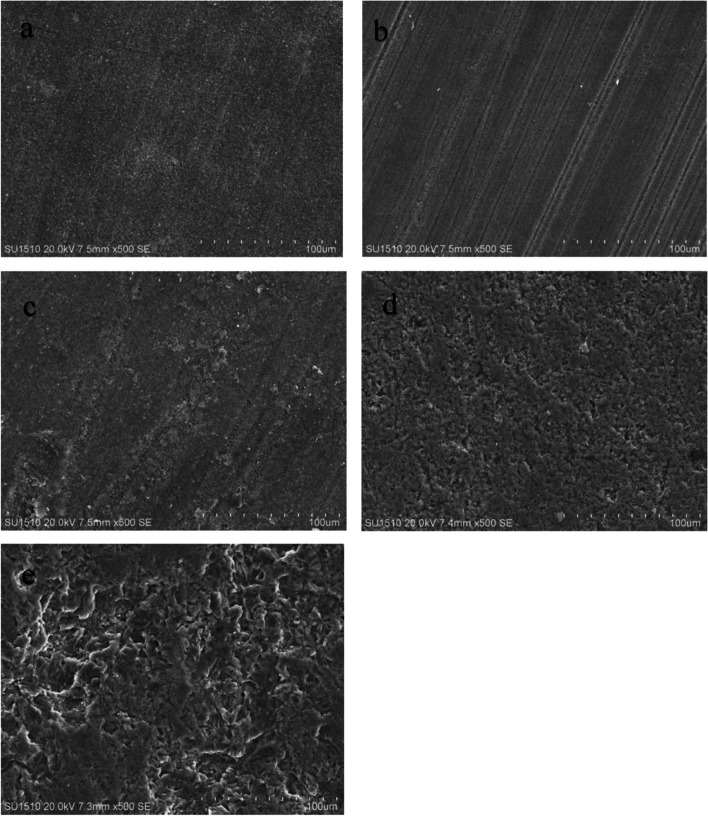
Scanning electron microscope images of specimens at fractured surface after biaxial flexural strength test (× 500) **a** Saremco, **b** Cerasmart, **c** Formlabs, **d** Vita Enamic, **e** Vita Mark II

Figure 2 shows SEM images of fractured specimens after BFS testing. The roughest surface was seen in the VM group followed by the VE group. In group CS, the smoothest SEM image was observed.

## Dıscussıon

The present study evaluated the effect of thermocycling on BFS and VHN of two 3D printed and two milled composite-based indirect restorative materials compared with a feldspathic glass-matrix ceramic. The first hypothesis of the study was accepted; the effect of thermal cycling was found to be insignificant on the BFS and VHN values of the materials. The secondary hypothesis of the study was rejected; the type of material had a significant effect on the BFS and VHN values of the permanent CAD-CAM indirect restorative materials.

Surface hardness, an important parameter to consider when comparing different materials, is the material's resistance to external nicks. Hardness, which is closely related to the wear resistance of the material, is one of the fundamental requirements of restorative materials, especially in the posterior stress bearing regions [27, 28]. In the present study, the microhardness values of the tested materials showed a statistically significant difference between materials, except for those that were 3D printed. VHN values of restorative materials ranged between 548.58 ± 11.27 Hv1 and 25.08 ± 0.45 Hv1. The material with the highest VHN values was VM (548.58 ± 11.27 Hv1), followed by VE (211 ± 9.61 Hv1). The materials with the lowest VHN value were FP (29.39 ± 0.53 Hv1) and SC (25.08 ± 0.45 Hv1) before thermocycling. Vitablocs Mark II is a feldspathic glass ceramic containing 20% feldspathic particles (4 mm-sized fine particles) and 80% glass matrix. The high VHN values of VM might be attributed to this composition. The difference between the VHN values of VE and CS, both of which were composite-based milled material, was statistically significant. VE had higher VHN values compared to CS. Cerasmart 270, referred to as a flexible nanoceramic, is a resin-matrix ceramic material that consists of relatively small and evenly distributed alumina-barium silicate particles embedded in a polymer matrix. It contains approximately 71% silica and barium glass nanoparticles and 29% composite resin by weight. Conversely, Vita Enamic is a hybrid material known as a polymer-infiltrated ceramic network. It typically consists of 86% feldspathic ceramic network and 14% polymer network by weight. The difference among VHN values of VE and CS might be attributed to the differences in composition. Brian et al. [29] found VM to have higher hardness than VE in their study. Grzebieluch et al. [4], in their examination of VHN, found a hardness of 273 Hv1 for Vita Enamic and 25 Hv1 for Varseo Smile Crown Plus 3D printing resin. In the study presented, the lowest VHN value was found in the 3D printed resin groups.

In a study conducted by Ellakany et al. [30] on temporary materials, the hardness value was 21 Hv1 in the SLA group and 16 Hv1 in the DLP group. In this study, when the VHN values were examined after thermal cycling, it was observed that VE and VM groups were affected. Husain et al. [31], in their examination of Saremco Print CrownTec before and after thermal cycling, found no change in VHN values, which supports the study presented.

The BFS of the materials were evaluated before the thermocycling process, and the highest BFS was observed in the CS group, followed by the 3D resin groups (FP then SC), and finally VE and VM. The feldspathic ceramic VM, which has been commercially available for several years, was chosen as the control group, due to its excellent aesthetic superiority and the required stable properties [32, 33]. However, in the study presented, VM showed the lowest BFS.

In parallel with the study presented, Grzebieluch et al. [4] found that the flexural strength of Varseo Smile Crown Plus 3D printed resin was better than Vita Enamic. After thermal cycling, a general decrease in BFS values was observed; however, no statistically significant difference was found. In a study conducted by Niem et al. [34] examining the effect of thermal cycling on the physical properties of different CAD-CAM restorative materials, the most durable material in terms of flexural strength after the thermal cycle was Cerasmart, followed by Vita Enamic and Vita Mark II, which also supports the study presented. When evaluating the BFS after the thermal cycle in the 3D printing group, it can be seen that FP (230.23 ± 32.60 MPa) was better than SC (215.31 ± 26.61 MPa). This difference can be attributed to the different production techniques of the resins used; SLA and DLP are 3D printing methods based on the principle of layer-by-layer formation using photocurable materials, known as vat polymerization [35]. In SLA, each layer is polymerized using ultraviolet laser light on a photoactive liquid resin, with the process repeated multiple times to achieve the final shape [36, 37]. This method is effective for complex geometries, but requires support structures during manufacturing and is relatively slow as it polymerizes a small area at a time. DLP systems also use a similar polymerization method to SLA [38]. However, in SLA, the ultraviolet light needs to scan the surface multiple times for complete polymerization, whereas DLP can polymerize the entire layer the first time [37, 39]. This is made possible by using a digital micro-mirror that transmits light and solidifies the resin at different positions within the layer [40]. These differences between methods can affect the mechanical properties. Ellakany et al. [30] conducted a study to investigate the influence of CAD-CAM milling and 3D printing fabrication methods on the mechanical properties of three-unit interim fixed dental prostheses after thermo-mechanical aging. The study found that among the 3D printing groups, the SLA group exhibited better flexural strength (167 MPa) compared with the DLP group (103 MPa). Türksayar et al. [41] in their study on temporary resin materials, reported that those produced by SLA had higher biaxial strength than those produced by DLP. In another study, samples obtained from temporary restoration materials produced by SLA technology exhibited a bending strength of 187.73 MPa, while DLP showed a lower bending strength of 153.51 MPa. Our results share the outcomes of these studies. Furthermore, in accordance with ISO 6872:2015 [39], adhesive cement single ceramic crowns are required to have a minimum flexural strength of 100 MPa, and the study presented found that all the tested materials met this minimum requirement [40].

SEM images of the fractured surfaces revealed differences between the materials. Depending on the microstructure variations, the fracture pattern and appearance of the fractured surfaces also differed. Among the fractured surfaces examined after thermal cycling, VM had the roughest surface appearance, which can also be attributed to the lowest BFS in this group. Following VM, VE had the second roughest surface appearance while the smoothest surface was observed in the CS group. It is believed that there is a correlation between surface roughness on SEM images and BFS [29].

The study had several limitations. First, it was an in vitro design that does not simulate saliva or chewing forces. Second, although material printing parameters and post-processing procedures adhered to manufacturers' protocols, the final polishing process was standardized to ensure uniform conditions for the tested materials. Third, this study used special liquids provided by manufacturers for use in 3D printers with various technologies for which there were differences in the composition of the liquids and printers used. However, as the printer range and liquid diversity increase, manufacturers have claimed that these liquids are compatible with different printers. Therefore, future research investigating different combinations will yield more comprehensive results.

Based on the findings of this study, differences in the composition and production techniques of the tested composite-based restorative materials led to different results on biaxial flexural strength and hardness. The tested 3D printed permanent composite resins showed BFS values between the BFS of the tested milled CAD-CAM composite materials and feldspathic ceramic, that were within the range of clinical acceptability. But hardness also plays a significant role in the long-term survival of indirect restorations. In clinical practice, 3D printed materials might not be appropriate in areas where posterior stress-bearing is significant. Therefore, more study is necessary to enhance the hardness needed for indirect restorations using 3D printed permanent composite resins made using various production procedures over the long term.

## Conclusıons

Within the limitations of the study; it was concluded that:

1. Among the tested materials, CS exhibited the highest BFS values, followed by the SC and FP groups produced with additive manufacturing. The VE and VM groups exhibited the lowest BFS values.
2. In the VHN test, VM exhibited the highest value, followed by VE and CS, respectively. Resin groups produced with additive manufacturing exhibited the lowest VHN values.
3. The thermal cycle had an insignificant effect on BFS values for all tested materials, while its effect on VHN values was significant only in the VE and VM groups.
4. Finally, permanent crown resins produced with 3D printers are successful for BFS results, but they need to be improved in terms of VHN values.

## Data Availability

The datasets used and analysed during the current study are available from the corresponding author on reasonable request.
